# Case Report: Recurrent meningioma with multiple metastases

**DOI:** 10.3389/fonc.2023.1192575

**Published:** 2023-07-17

**Authors:** Juyue Zhou, Zhonghai Du

**Affiliations:** ^1^Graduate Institute, Shandong University of Traditional Chinese Medicine, Jinan, Shandong, China; ^2^Department of Oncology, Weifang Hospital of Traditional Chinese Medicine, Weifang, Shandong, China

**Keywords:** case report, meningioma, recurrence, aggressiveness, extracranial metastasis

## Abstract

Post-surgery recurrence of meningiomas with multiple extracranial metastases is rare. Currently, information on extracranial metastases is limited, and no clear predictors and standardized treatment protocols can be applied clinically. Herein, we report a case of meningioma that recurred after two surgeries and had multiple distant metastases. Computed tomography revealed multiple enlarged lymph nodes in the para-aortic arch, left lower lung region, retroperitoneum, and abdominopelvic region, as well as soft tissue mass-like lesions under the liver capsule in the right lobe of the liver. Magnetic resonance imaging showed space-occupying lesions under the cranial plate of the left parietal lobe. Tissue biopsy confirmed the diagnosis of recurrent meningioma with extracranial metastases. Immune checkpoint inhibitors and anti-angiogenic drugs were administered. After two treatment cycles, the patient’s clinical symptoms were significantly relieved, and the imaging assessment confirmed a stable disease. Although it did not meet our expectations, this combination therapy still demonstrated a possible benefit in improving meningioma patients’ survival and quality of life. In this report, along with the case, we also review the relevant literature on the subject and discuss the associated risk factors and treatment options.

## 1 Introduction

As brain tumors originate from arachnoid cap cells of the leptomeninges, most meningiomas are benign (1, 2). Psammoma bodies—which exhibit concentric calcification, nuclear pseudoinclusions, pseudo-syncytial growth, and tumor cells forming whorls—serve as the basis for pathological diagnosis (3). In immunohistochemistry, the somatostatin receptor 2 (*SSTR2*) and progesterone receptor (*PR*) can be used as diagnostic markers (4). For accurate clinical diagnosis, the World Health Organization (WHO) divides meningiomas into three grades based on mitotic activity, degree of brain invasion, or particular histological characteristics (5). Ninety percent of meningiomas are WHO grade 1, histologically characterized as meningothelial, fibroblastic, transitional, secretory, psammomatous, metaplastic, microcystic, or angiomatous (6). Krüppel-like factor 4(*KLF4*)/TNF receptor-associated factor 7(*TRAF7*) mutations can also be used to diagnose secretory meningiomas (5). Clear cells, chordoid variants, and atypical meningiomas are categorized as WHO grade 2 atypical meningiomas (5, 6). Notably, the presence of brain invasion satisfies the grade 2 criteria (6). WHO grade 3 meningiomas (anaplastic meningiomas) are the rarest type (5, 6). According to the latest grading criteria, meningiomas should be classified as WHO grade 3 whenever telomerase reverse transcriptase (*TERT*) promoter mutations and/or cyclin-dependent kinase inhibitor 2 A/B(*CDKN2A/B*) pure deletions are present (5). Grade 1 meningiomas often recover after total excision, with few distant metastases (7). By contrast, grade 3 meningiomas have significant rates of recurrence and metastasis, with the lungs, bones, spinal cord, and liver being the most frequent sites (8–10). WHO grade 2 tumors are less malignant than grade 3 tumors, rarely have associated metastases, and exhibit extracranial metastasis much less frequently. In this report, we describe the case of a patient with several metastases from a recurrent meningioma following surgery and evaluate the relevant literature on the subject.

## 2 Case description

**Figure 1:**
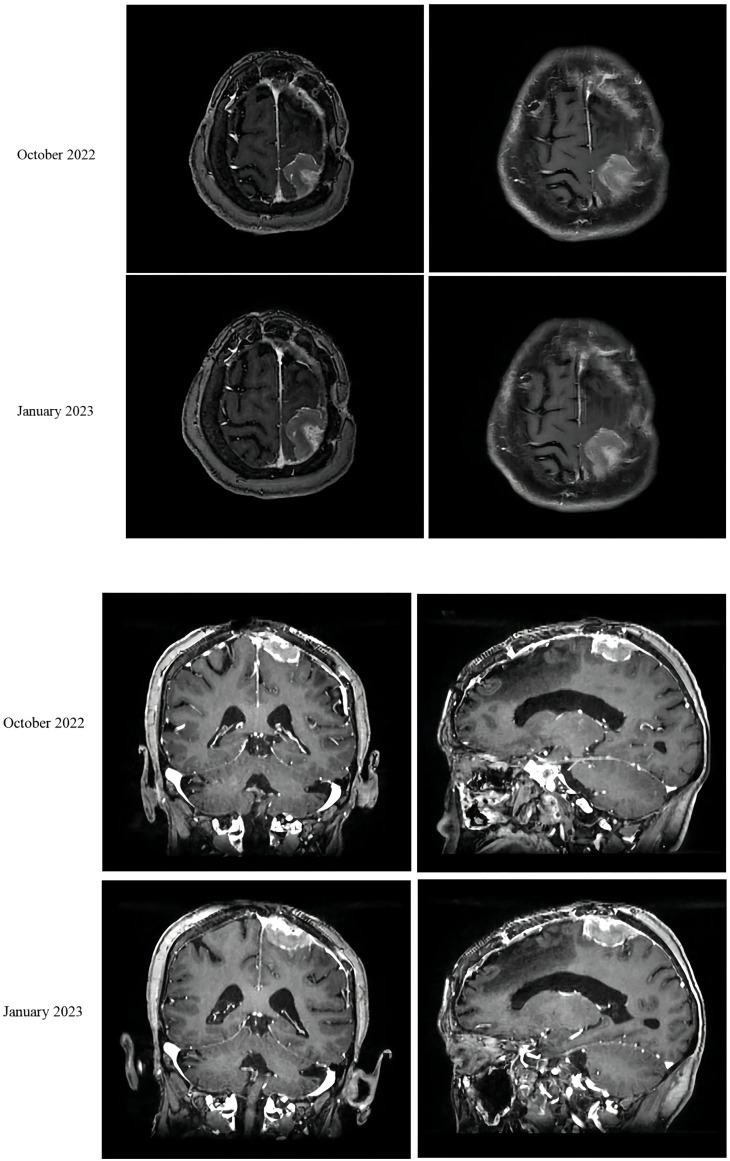
MRI of the patient’s meningioma in October 2022 and January 2023.

**Figure 2:**
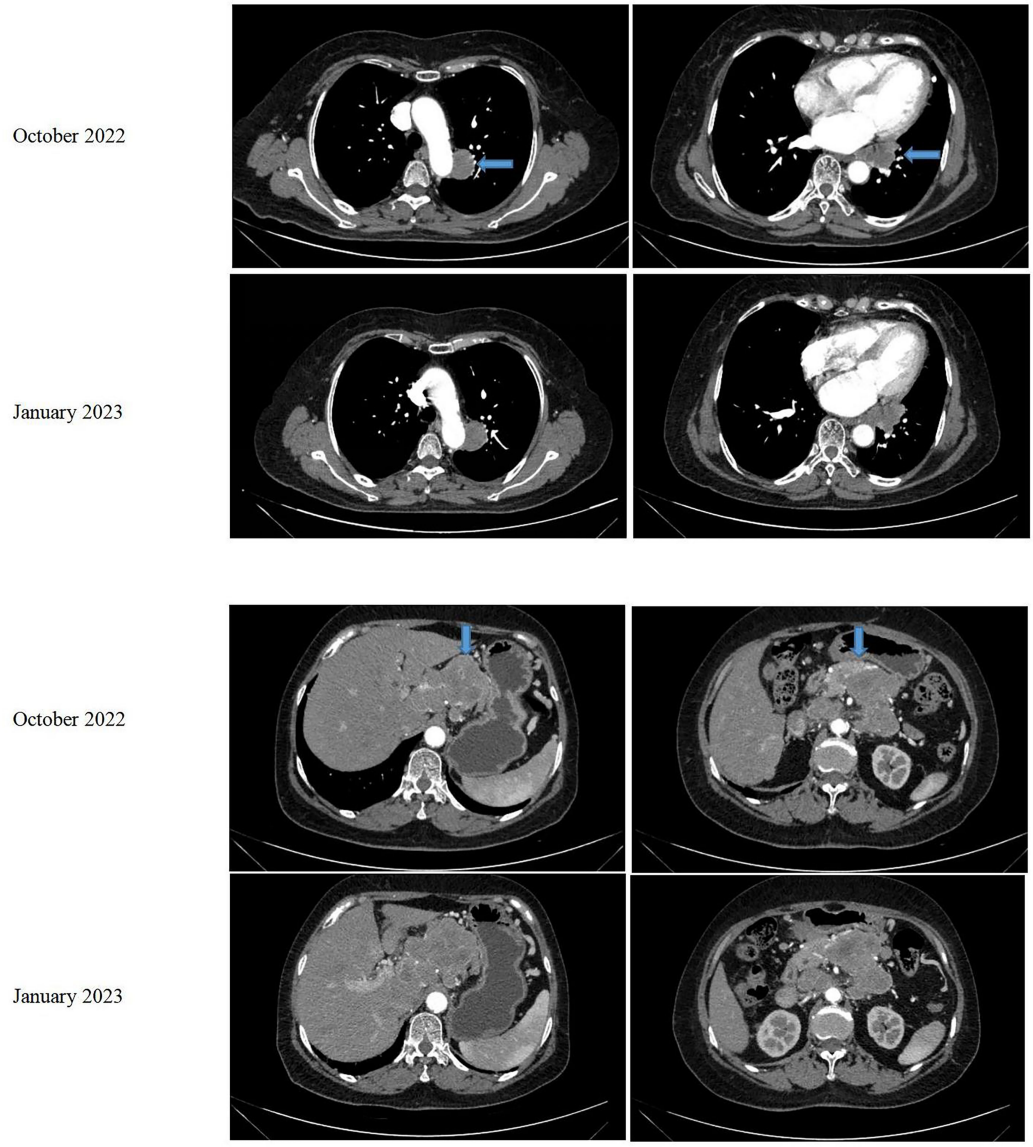
CT of the patient’s chest and abdomen in October 2022 and January 2023.

In August 2021, a 68-year-old female patient was brought to our hospital with sudden unconsciousness and limb convulsion. Upon magnetic resonance imaging (MRI), a space-occupying lesion compressing the brain tissue was discovered in the left frontal lobe, adjacent to the inner plate of the skull. The patient had previously undergone surgical treatment for a WHO grade 1 meningioma in 2017. There was no other treatment after the surgery, and the patient was managed mainly through observational follow-up. Two weeks later, the patient underwent complete tumor resection (Simpson grade I). The postoperative pathological result was a WHO grade 2 meningioma. No follow-up treatment was performed after this surgery. The grade evaluation of meningiomas in 2017 and 2021 were based on the histopathological assessment of post-surgical biopsy specimens and did not utilize molecular testing. However, she returned to our hospital in October 2022, complaining of abdominal pain. An MRI revealed space-occupying lesions in the left parietal lobe underneath the cranial plate (**Figure 1**). A computed tomography (CT) scan revealed several enlarged lymph nodes in the left lower lung area, retroperitoneum, and abdominopelvic region, as well as soft tissue mass-like lesions under the capsule of the right lobe of the liver (**Figure 2**).

**Figure 3:**
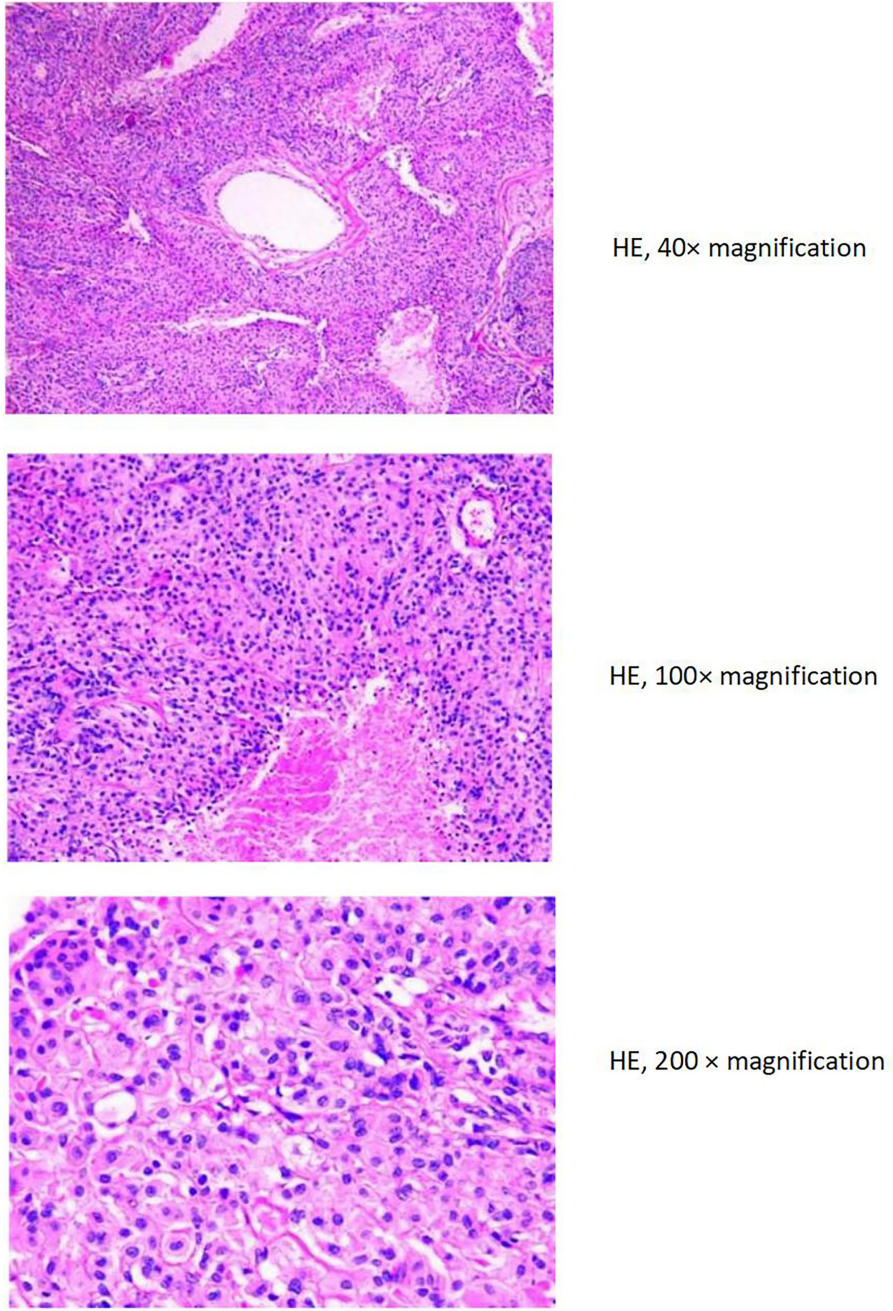
Hematoxylin & eosin stain of tumor biopsy specimens in 2022.

**Figure 4:**
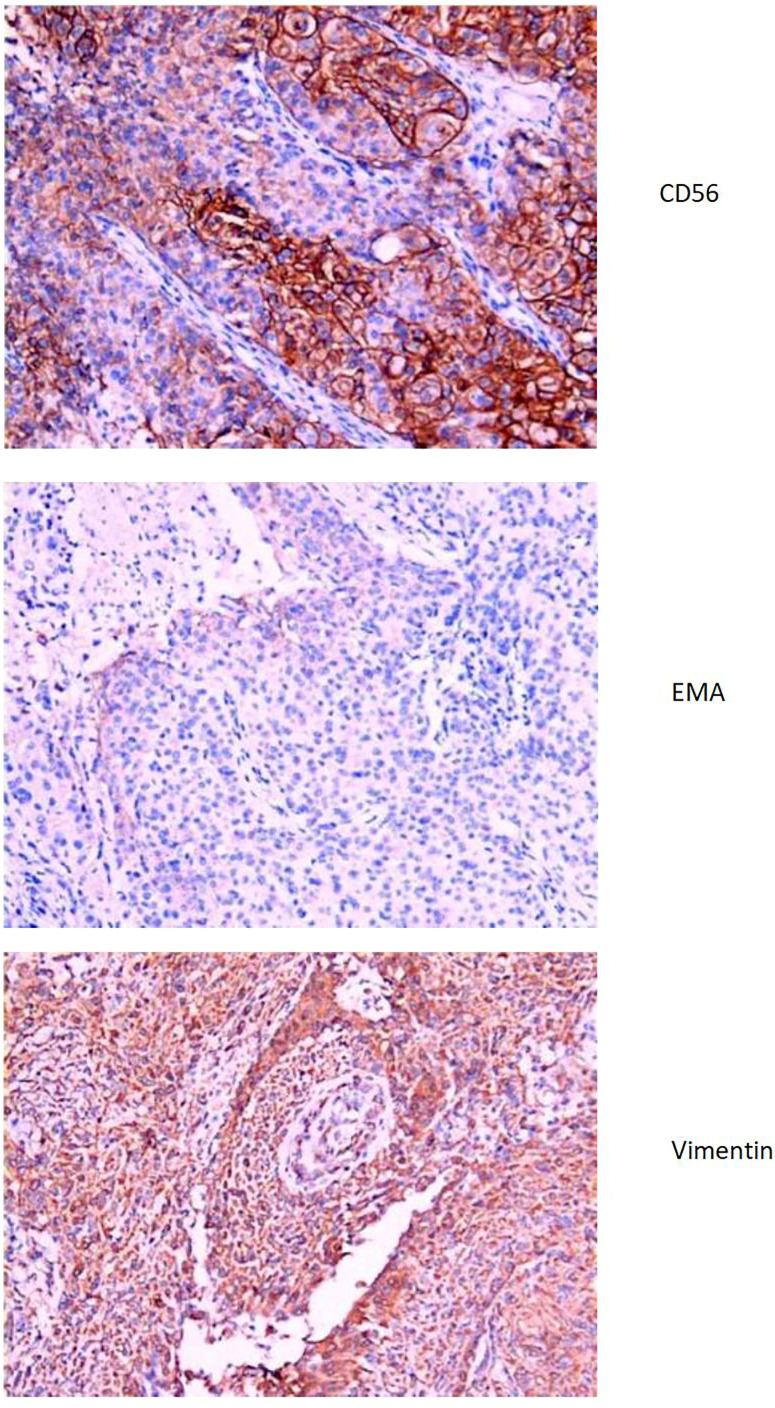
Immunohistochemistry of tumor biopsy specimens in 2022. CD56(+), EMA(+), Vimentin(+).

The liver and abdominal lymph nodes were biopsied, and metastatic meningiomas were confirmed by pathological evaluation. Histopathology suggested that the tumor cells were densely arranged, and epithelial-like, with a large nucleus, increased nucleoplasm ratio, and prominent nucleoli. And there were more than 4 mitotic figures per 10 high power fields and sheets of tumor cell necrosis (**Figure 3**). The pathological specimens tested positive for CD56, Vimentin, Desmin, S-100, EMA but were negative for CK, CD34, NSE, PR, MelanA, α-inhibin, and PD-1(**Figure 4**). The composite positive score of programmed cell death-ligand 1(PD-L1) was approximately 5%. Next-generation sequencing(NGS) detection revealed an *NF2* mutation, 1p deletion, *CDKN2A* deletion, *CDKN2B* deletion, and no abnormalities in *TERT*. The genotype of *NF2* was intron2 c. 241-9A>G with 76.38% mutation abundance. These test results suggested that the patient had a recurrence of meningioma, which became a grade 3 malignancy with multiple metastases. A combination therapy of anti-programmed death 1 (PD-1) and anti-vascular endothelial growth factor (VEGF) was administered after the patient, and the family agreed to the treatment. After two treatment cycles, the patient’s clinical symptoms were significantly relieved, and the imaging assessment confirmed a stable disease (**Figures 1**, **2**). The patient is still on this regimen, and we will continue to monitor the patient’s condition based on assessments every two treatment cycles.

## 3 Discussion

This report describes an unusual case of postoperative meningioma recurrence with several metastases. The recurrence rate of WHO grade 2 meningiomas ranges from 40% to 50% within ten years of surgery. The variables influencing recurrence risk include tumor grade, postoperative radiation, postoperative tumor volume, low apparent diffusion coefficient (ADC), Simpson’s resection grade, and repeat craniotomy (11–14). The subgroup of meningiomas with *NF2*, *PI3K*, *HH*, and *TRAF7* alterations is more prone to recurrence, with recurrence in the NF2 subgroup being associated with sex, tumor grade, and Ki-67 (15). Furthermore, differences in genetic signature scores and molecular subgroups are associated with shorter times to recurrence. In addition, miR- 146a-5p and miR-331-3p in microRNAs can be useful in predicting recurrence (16). Our patient underwent multiple craniotomies, which may have predisposed her to a greater risk of recurrence. The remaining potential predictors were not present in her case.

Metastases are primarily observed in patients with WHO grade 3 meningiomas (9). High cellularity, cellular heterogeneity, rate of mitosis, nuclear pleomorphism, tumor necrosis, and invasion of nearby vessels are the reported predictive factors (9). A retrospective chart review of 149 patients revealed that those with atypical and anaplastic meningiomas with scalp infiltration were more likely to experience systemic metastases (17). In a study based on the SEER database, 133 patients had distant metastases, representing 0.18% of the study population, and tumor volume, male gender, and WHO grades may be factors for distant metastases (18). One case report on atypical meningioma with pulmonary metastases suggested that patients with local tumor recurrence or incomplete resection, with the tumor close to the venous sinus and chromosomal instability, should be followed up for early detection of metastases (19). People with a history of meningioma should be given special consideration when diagnosing a new mass, even if the tumor is in complete remission after surgery or if there has been a long interval from this disease (20, 21). Tissue biopsy remains the gold standard for diagnosis because distant metastases are challenging to identify using imaging alone.

There are no clear treatment criteria for progressive or metastatic meningiomas. The recommended drugs include small-molecule protein kinase inhibitors, anti-angiogenic drugs, immune checkpoint inhibitors, and peptide receptor radionuclide therapy, among others (22, 23).

We selected an immune checkpoint inhibitor and an anti-angiogenic medication to treat the recurrent metastatic meningioma. VEGF has been demonstrated to play a significant role in controlling angiogenesis in meningiomas and being a necessary component of tumor growth (24, 25). The expression level of VEGF correlates with the WHO classification and is higher in higher-grade meningiomas (26). Many clinical trials have evaluated sunitinib as a therapeutic agent (27). Kaley et al. used sunitinib in patients with WHO grade 2-3 recurrent meningioma and reported a median progression-free survival (PFS) of 5.2 months and a median overall survival(OS) of 24.6 months (28). They also reported more prolonged survival in patients harboring tumors positive for VEGF expression (26). Retrospective studies have indicated that bevacizumab effectively treats recurrent meningioma (29, 30). A prospective analysis of 17 patients with recurrent meningiomas revealed a median PFS of 22 months after combined treatment with bevacizumab and everolimus (31). However, the results may have been influenced by the 29% of patients who had good prognosis grade 1 meningiomas. Bevacizumab also exerts an anti-edema effect that can lessen edema around the tumor by decreasing vascular permeability through inhibiting VEGF expression (30, 32).

Due to their dura-based anatomical position, absence of the blood-brain barrier, and suppressed immunological milieu caused by high PD-L1 expression—linked to poor prognosis—meningiomas are more likely to experience tumor recurrence or progression (33, 34). Research on meningioma immunotherapy has revealed that pembrolizumab can treat aggressive or progressive meningiomas, including those with distant metastases, with a median PFS of 7.6 months and median OS of 20.2 months (35). Nivolumab treatment led to a median PFS of 5.56 months in patients with recurrent meningiomas, and the median OS at 12-month follow-up was outstanding at 30.93 months (23). Thus, immune checkpoint inhibitors are worthwhile alternatives for meningioma treatment. However, more clinical evidence is required to support this option.

Although the expected outcome was not achieved in our case, currently, our patient has a stable disease, which suggests that a treatment regimen combining immunotherapy with anti-angiogenic drugs may benefit the survival of meningioma patients undergoing recurrence.

## 4 Conclusion

We describe the case of a female patient with meningioma who experienced recurrence along with distant metastases following numerous surgeries. Extracranial metastases from meningiomas are uncommon and may more likely occur in individuals who have undergone multiple brain tumor surgeries in the past. Meningiomas that are close to the venous sinuses, highly malignant meningiomas, or those with chromosomal instabilities detected in the primary tumor are also more likely to undergo metastasis. Pathological examination is necessary for patients with a history of meningioma who present with unidentified masses at other sites. Anti-angiogenic drugs and immune checkpoint inhibitors have shown effectiveness in patients, and further studies of this combination therapy are needed.

## Data availability statement

The original contributions presented in the study are included in the article/supplementary material. Further inquiries can be directed to the corresponding author.

## Ethics statement

Written informed consent was obtained from the individual(s) for the publication of any potentially identifiable images or data included in this article.

## Author contributions

All authors contributed to patient care. JZ summarized the clinical data and wrote the manuscript, and ZD collected the clinical data. All authors participated in the revision of the article and approved the submitted version.
